# Systemic lupus erythematosus with acute ischemic optic neuropathy complicated with neuromyelitis optica: a case report

**DOI:** 10.1186/s13256-022-03734-8

**Published:** 2023-01-22

**Authors:** S. K. Jakaria Been Sayeed, Asif Hasan Khan, Md. Moniruzzaman, Reaz Mahmud, Md. Mujibur Rahman

**Affiliations:** 1 Clinical Neurology, National Institute of Neurosciences and Hospital, Dhaka, Bangladesh; 2 Department of Neurology, Dhaka Medical College Hospital, Dhaka, Bangladesh; 3 Department of Medicine, Bangabandhu Sheikh Mujib Medical University, Dhaka, Bangladesh

**Keywords:** Neuromyelitis optica, Systemic lupus erythematosus, Acute ischemic optic neuropathy

## Abstract

**Background:**

Neuromyelitis optica is a relapsing–remitting disease characterized by a recurrent attack of optic neuritis and transverse myelitis; sometimes associated with acute brainstem syndrome. Systemic lupus erythematosus is an autoimmune multisystem disorder in which ocular involvement such as acute ischemic optic neuropathy is a rare manifestation. However, neuromyelitis optica can be associated with systemic lupus erythematosus.

**Case presentation:**

A 24-year-old Bangladeshi woman was admitted to the hospital with complaints of sudden, progressive, painless vision loss in both eyes, and progressive weakness in both lower limbs for 48 hours. She also gave a history of arthralgia, a photosensitive skin rash, intermittent fever, oral ulcerations, and alopecia for the last 2 months. On examination, the fundus was suggestive of bilateral acute ischemic neuropathy, and examinations of the lower limb revealed spastic paraparesis with sensory abnormality. Laboratory investigations revealed the presence of positive anti-aquaporin 4 antibody, strongly positive antinuclear antibody, and anti-ds DNA with the longitudinally extensive lesion on magnetic resonance imaging of the spinal cord. She was treated with methylprednisolone, hydroxychloroquine, and mycophenolate, and was discharged with improvement of her paraparesis. However, her vision did not improve substantially.

**Conclusion:**

The importance of this report is to shed some light on the occurrence of two devastating complications that is, bilateral acute ischemic optic neuropathy in systemic lupus erythematosus complicated by neuromyelitis optica, as well as evidence of rare presentations for systemic lupus erythematosus and treatment modalities of ischemic optic neuropathy with systemic lupus erythematosus.

## Introduction

Neuromyelitis optica (NMO) is an aggressive inflammatory disorder characterized by recurrent attacks of optic neuritis and longitudinally extensive transverse myelitis, sometimes associated with additional central nervous system structure involvement such as area postrema syndrome, diencephalic syndrome, and so on. Therefore, NMO has been defined recently as NMO spectrum disorder (NMOSD; previously known as Devic’s disease) [1]. The majority of NMOSD is associated with anti-aquaporin 4 antibody (AQ4 Ab) [2]. Sequential or concomitant attacks of transverse myelitis and optic neuritis, with contiguous spinal cord lesions on magnetic resonance imaging (MRI) extending over three or more vertebral segments, with the presence of anti-aquaporin 4 antibodies (AQ4 Ab) distinguish NMOSD from multiple sclerosis [3]. NMO patients do have associations with systemic autoimmune disorders such as systemic lupus erythematosus, Sjogren’s syndrome, mixed connective tissue disease, antineutrophil cytoplasmic antibody (ANCA)-associated vasculitis, and myasthenia gravis [4, 5]. Systemic lupus erythematosus (SLE) is an autoimmune disorder involving multiple organ systems such as musculoskeletal, skin, kidney, lungs, hematological, central, and peripheral nervous systems [6]. NMO with SLE has rarely been reported because the chances of a patient having both SLE and NMO are 1 in 5,000,000 [7, 8], and a pathophysiological link between SLE and NMO has not yet been completely established [9]. However, it has been reported previously that NMO can be the first presentation of SLE [10]. Optic nerve disease, represented by optic neuritis and ischemic optic neuropathy (anterior and posterior) is a rare, but more severe, vision-threatening manifestation that affects only 1% of SLE patients [11]. Specific clinical features with laboratory investigations such as retinal photography and cerebrospinal fluid (CSF) evaluation to identify the absence of the oligoclonal band, and the presence of distinctive longitudinally extensive transverse myelitis, anti-aquaporin 4 antibody (AQ4 IgG Ab), and associated autoimmune markers are necessary to establish the diagnosis of NMOSD [12]. Here, we report a case of SLE patient who had arthralgia, skin rash, oral ulceration, and alopecia for 2 months, followed by sudden onset of painless vision problems with weakness in both lower limbs. The importance of this report is to shed light on the occurrence of two devastating complications: bilateral acute ischemic optic neuropathy in SLE complicated by NMO, a very rare presentation for SLE.

## Case presentation

**Fig. 1:**
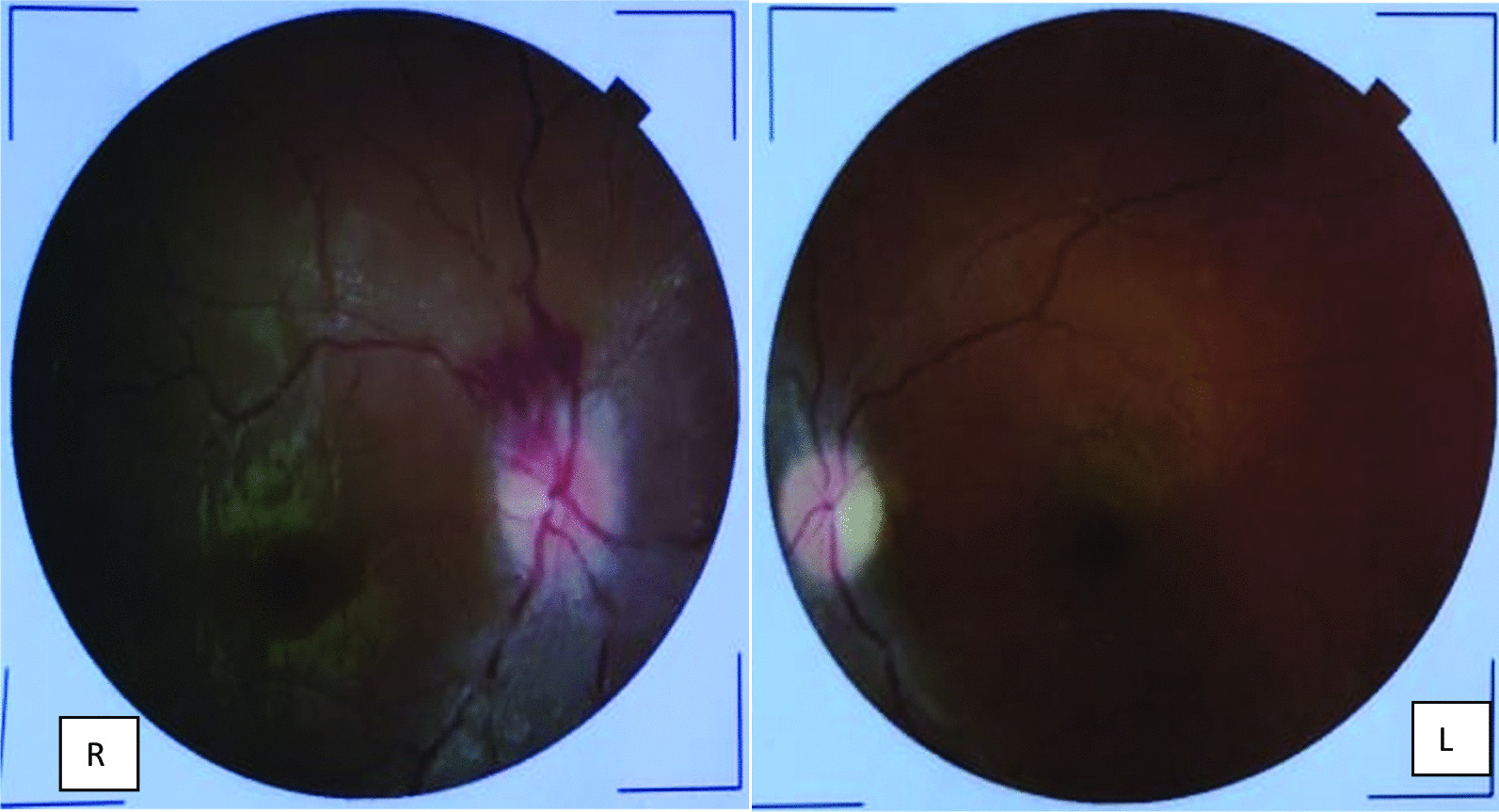
Retinal photograph showing bilateral pale and swollen optic disc with flame-shaped hemorrhage at the superotemporal part of right optic disc suggestive of bilateral acute ischemic optic neuropathy. (R, right; L, left)

**Fig. 2:**
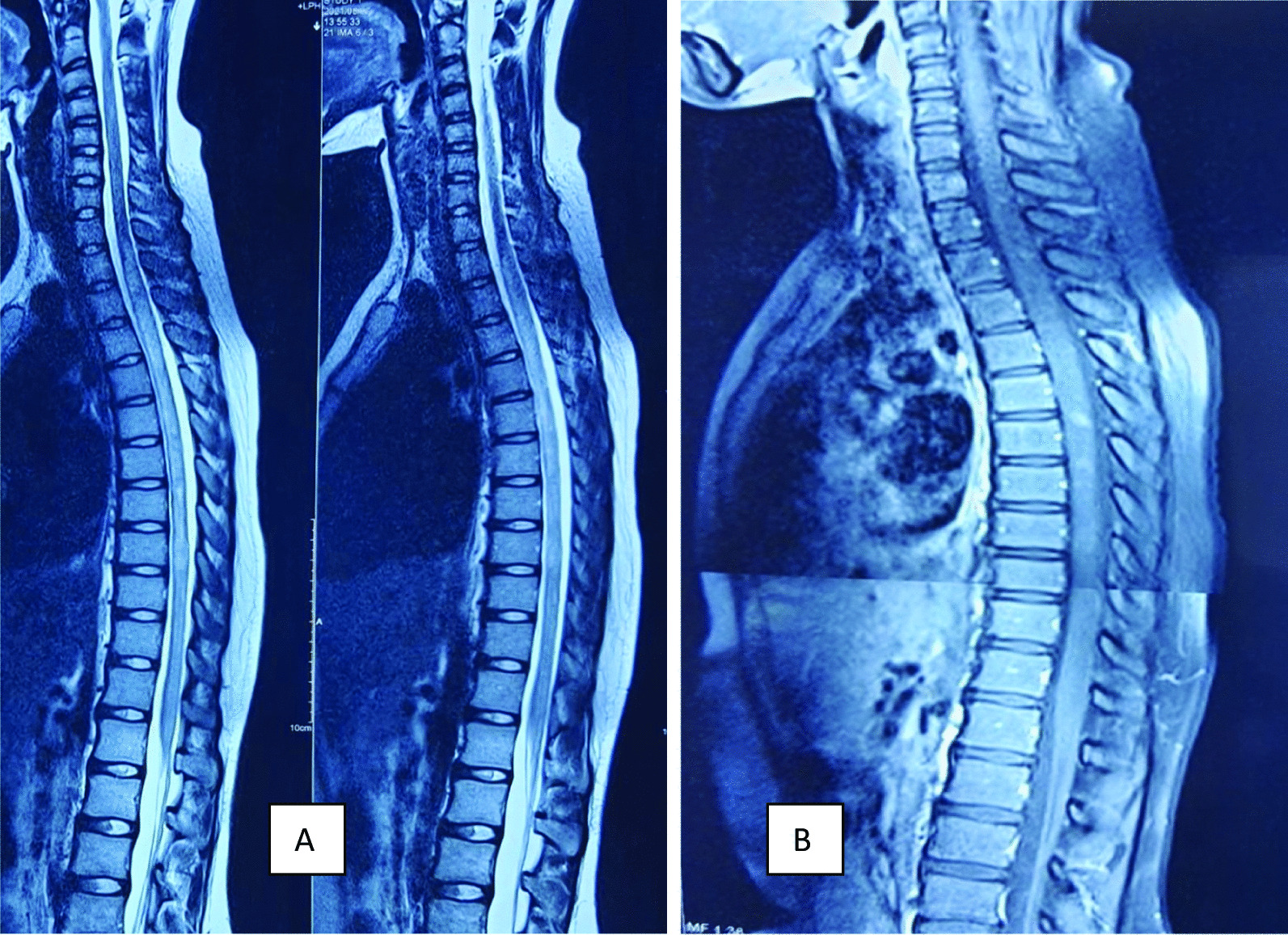
**A**, **B** MRI of spinal cord with contrast revealed multiple enhancing T2W1 hyperintense areas only in cervical and dorsal spinal cord with diffuse cord edema

We report the case of a 24-year-old Bangladeshi woman with a 2-month history of polyarthralgia, photosensitive skin rash, intermittent fever, oral ulcerations, and alopecia. The patient was admitted to our hospital with complaints of sudden, progressive, painless vision loss in both eyes, and sudden, progressive weakness in both lower limbs for 48 hours. There was no history of painful skin rash over limbs, Raynaud’s phenomenon, digital gangrene, headache, seizure, bowel and bladder problems, first-trimester abortion, or trauma to the back or neck region. Her family history was unremarkable and she had not suffered from a similar type of illness before. On examination, her vitals were normal, malar rash over her face, alopecia, oral ulceration, and small joints including wrist joints of both hands were swollen and tender. However, skin tightness and digital ischemia were absent. Visual acuity was reduced in both eyes to the perception of hand movement. Projection of light and projection of rays were present and direct light reflex was sluggish with normal consensual reflex in both eyes. Intraocular pressure measured by tonometer in both eyes was 15 mmHg (normal range 11–21 mmHg). Fundoscopic examination revealed bilateral pale and swollen optic discs with a flame-shaped hemorrhage at the superotemporal part of the right optic disc suggestive of acute ischemic optic neuropathy (Fig. 1). There was bilateral upper motor neuron type weakness in both lower limbs evidenced by reduced muscle power and brisk knee and ankle jerk with plantar extensor. There was a reduced sensation (pain, temperature, and joint position) and indications of sensory deficit up to the thoracic eight segments of the spinal cord. Other systemic examinations were unremarkable. Laboratory findings revealed normocytic normochromic anemia, positive anti-aquaporin 4 antibody (AQ4 IgG Ab), strongly positive antinuclear antibody (ANA) (homogenous), a high titer of anti-ds DNA, and low complement level (C3 and C4). CSF study was unremarkable including oligoclonal bands, which were absent (Table 1). MRI of the brain, orbit, and spinal cord with contrast revealed multiple enhancing T2W1 hyperintense areas only in the cervical and dorsal spinal cord with diffuse cord edema; the brain and orbit were unremarkable. Magnetic resonance venogram (MRV) with contrast was unremarkable (Fig. 2).

**Table 1:** Laboratory findings of patient on admission

Trait	Value	Reference
Hemoglobin	9.6 g/dL	11.5–15.5 g/dL
White blood cells	5000 mm^3^	4–11,000 mm^3^
Platelets	155,000 mm^3^	150,000–4,500,000 mm^3^
CRP	3.12 mg/L	< 6 mg/L
MCV	78 fL	76–96 fL
MCH	29 pg	27–32 pg
MCHC	30 g/dL	30–35 g/dL
PBF	Normocytic normochromic anemia	
Urine R/E	Unremarkable	
S. creatinine	0.66 mg/dL	0.5–1.2 mg/dL
SGPT	23 IU	10–40 IU
D-dimer	0.35 g/L	< 0.5 g/L
APTT	34 seconds	32–40 seconds
ANA	Strongly positive, homogenous pattern on immunofluorescence	
Anti-ds-DNA	185 Iu/mL (strongly positive)	< 30 Iu/mL
Anticardiolipin Ab		
IgM	2.35 U/mL	< 15 U/mL
IgG	3.9	
Antibeta 2 glycoprotein 1 antibody		
IgM	7.9	
IgG	8.5 U/mL	< 40 U/mL
Complement		
C3	0.07 g/L	0.9–1.8 g/L
C4	0.03 g/L	0.2–0.5 g/L
p-ANCA	1.91 U/mL (negative)	< 5 U/mL
c-ANCA	2.32 U/mL (negative)	< 5 U/mL
S. TSH	1.98 μIu/mL	0.85–4.54 μIu/mL
FT4	1.12 ng/dL	0.7–1.48 ng/dL
CSF study	Appearance—clear	
	Cell count—lymphocyte 3 cumm	0–5 cumm
	Protein—35.4 mg/dL	15–45 mg/dL
	Glucose—3.42 mmol/L	2.2–3.9 mmol/L
	Oligoclonal band—absent	
	Gram, AFB stain and culture—negative	
S. anti-NMO Ab	35.01 μ/mL	< 3 μ/mL (no detectable antibody)
S. electrolyte		
Na^+^(sodium)	137 mmol/L	135–145 mmol/L
K^+^ (potassium)	3.9 mmol/L	3.5–5.5 mmol/L
Mg^2+^ (magnesium)	1.89 mmol/L	1.7–2.2 mmol/L
Ca^2+^ (calcium)	10.2 mg/dL	9–11 mg/dL

*APTT* activated partial thromboplastin time, *ANA* antinuclear antibody, *ANCA* antineutrophilic cytoplasmic antibody, *anti-NMO Ab* antineuromyelitis optica antibody, *CRP* C-reactive protein, *CSF* cerebrospinal fluid, *PBF* peripheral blood film, *TSH* thyroid-stimulating hormone

Immediately after clinical examination, intravenous methylprednisolone 1 gm once daily for 5 days was started, followed by 60 mg prednisolone once, hydroxychloroquine 300 mg, and 2000 mg mycophenolate mofetil per oral daily. After taking methylprednisolone, her vision did not improve substantially (hand movement to finger counting). However, muscle power did improve (2/5 to 4/5) in both legs in 2 weeks and she could walk by herself like a normal person. She has been kept under regular follow-up by a multidisciplinary team.

## Discussion

SLE is a multisystemic disorder that has a very complex pathogenesis comprising multiple factors including gender and environment. These factors cause the formation of both antibodies and immune complexes, which can lead to a systemic, global inflammatory reaction involving multiple organs [6]. In 2019, European Alliance of Associations for Rheumatology/American College of Rheumatology (EULAR/ACR) classified SLE based on positive ANA as an obligatory criterion; in addition to seven clinical (constitutional, hematologic, neuropsychiatric, mucocutaneous, serosal, musculoskeletal, renal) and three immunological (antiphospholipid antibodies, complement proteins, SLE-specific antibodies) domains, and clarified a scoring system of a “weight from 2 to 10.” A patient who has ≥ 10 points will be classified as SLE [13]. Our patient scored 24 points according to the updated criteria. Occular involvement such as keratoconjunctivitis sicca, iridocyclitis, retinal vasculitis, vasoocclusive disease, choroidopathy, and optic neuropathy can be presenting features of SLE [14]. However, it is worth mentioning that there are some differences between SLE-associated optic neuritis and optic neuropathy. Optic neuritis is characterized by the acute unilateral painful loss of vision due to infarction of the optic nerve secondary to arteriolar fibrinoid necrosis, whereas optic neuropathy associated with SLE presents as bilateral, painless vision loss, with or without optic disc swelling due to an ischemic process affecting the optic nerve head and retrobulbar nerve [14, 15]. For SLE-induced optic neuritis or optic neuropathy, advanced testing such as fundus fluorescein angiography (FFA), visual field test, and MRI of orbit with gadolinium scan can be done for differentiation between the diseases [11, 16]. Our patient presented with bilateral acute painless loss of vision with optic disc swelling that was in favor of ischemic optic neuropathy related to SLE-induced vasculitis. In addition, our patient suffered the first attack of transverse myelitis, which was clinically more suggestive of NMO, evidenced by the presence of highly specific anti-NMO IgG antibodies in high titer along with the longitudinally extensive lesions in both cervical and dorsal spinal cord. Moreover, paraparesis and sensory abnormality improved after giving methylprednisolone; however, her vision did not improve substantially. Mehta *et al*. [10] reported a case of SLE where the patient suffered recurrent transverse myelitis without optic neuritis due to NMO, evidenced by positive anti-NMO-antibody and longitudinally extensive lesion in the spinal cord. Sequential or concomitant attacks of transverse myelitis and optic neuritis, with contiguous spinal cord MRI lesions extending over three or more vertebral segments with the presence of anti-aquaporin 4 antibodies (AQ4 Ab) not only define NMOSD but also differentiate it from multiple sclerosis [3, 12]. Relapsing–remitting multiple sclerosis (RRMS) is a strong differential of NMOSD. Sometimes it is difficult to distinguish both diseases by clinical features only. A typical MRI pattern of multiple sclerosis is at least one lesion on a T2-weighted scan (which could be T2 spin echo or fluid-attenuated imaging) in both the inferior temporal lobe and adjacent to the lateral ventricle, or either a subcortical lesion with a U-fiber-type morphology (s-shaped or curved) or an ovoid lesion perpendicular to the lateral ventricle (Dawson’s fingers). In the spinal cord, the lesion will be a short segment. This radiological criterion has 92% sensitivity and 96.2% specificity. On the contrary, the presence of anti-aquaporin 4 antibodies with longitudinally extensive lesions in the spinal cord (T2 and FLAIR) goes more in favor of NMOSD [17]. It is clear that our patient had active SLE [fever, oral ulceration, arthralgia, raised erythrocyte sedimentation rate (ESR), high titer anti-ds-DNA, low C3 and C4, and ischemic optic neuropathy] but it is very unlikely that SLE causes longitudinally extensive spinal cord lesion and anti-NMO antibody. That is why we considered NMO as the cause of typical MRI lesions in the spinal cord. To the best of our knowledge, this is the first case in Bangladesh where SLE with acute ischemic optic neuropathy (AION) was associated with NMO; which is extremely rare. For SLE-associated ocular disease, immunosuppressive medications are the mainstay of treatment [18]. High-dose intravenous methylprednisolone (1 g/day for 3 days) followed by oral prednisone (1 mg/kg/day) is the first line of treatment for SLE-associated optic neuropathy [19]. Currently, the first-line therapy for severe NMO is azathioprine or rituximab, second-line therapy is azathioprine to rituximab or vice versa, or mycophenolate mofetil, methotrexate, or mitoxantrone in case of side effects or poor response. However, third-line therapy tocilizumab should be started if disease progression occurs and/or the above treatments fail [20]. In our patient, intravenous methylprednisolone 1 gm was given for five consecutive days followed by oral prednisolone 60 mg per day, hydroxychloroquine 300 mg per day, and mycophenolate mofetil 2 gm per day. The outcome of optic neuropathy related to SLE is variable. Jabs *et al*. [21] described that four of their seven patients partially improved following treatment with corticosteroids. Rosenbaum mentioned in their observational study that four patients suffered SLE-associated ischemic optic neuropathy; visual acuity improved only in two of them on intravenous cyclophosphamide therapy because of early presentations and young age [22]. In our patient, visual acuity did not improve adequately, probably because of late presentation (more than 96 hours) and not receiving cyclophosphamide. We believe that early initiation of intravenous cyclophosphamide in conjunction with methylprednisolone is the best treatment option for neuro-ophthalmic manifestations of SLE.

## Conclusions

Neuro-ophthalmic manifestations of SLE are the most devastating and extremely rare presentations. In addition, the presence of NMO in the same patients might cause more complications and variable outcomes. Therefore, it should be distinguished first in SLE patients whether the vision problem is related to optic neuritis or ischemic optic neuropathy, is related to SLE, NMO, or MS, because treatment modalities and prognosis differ. Early initiations of cyclophosphamide and methylprednisolone might prevent vision loss in SLE-associated optic neuropathy even though cyclophosphamide is not recommended for NMO if vision problems are related to NMO.

## Data Availability

Not applicable.

## Abbreviations

AION: Acute ischemic optic neuropathy
ANCA: Antineutrophil cytoplasmic antibody
ANA: Antinuclear antibody
CSF: Cerebrospinal fluid
C3 and C4: Complement 3 and 4
CRP: C-reactive protein
NMO: Neuromyelitis optica
SLE: Systemic lupus erythematosus
TSH: Thyroid-stimulating hormone
FT4: Free thyroxin
MRV: Magnetic resonance venogram
